# A matter of time: Cardiac aneurysm and recurrent thromboembolic events

**DOI:** 10.1007/s12471-026-02031-z

**Published:** 2026-03-04

**Authors:** Luísa Pinheiro, Margarida de Castro, Emídio Mata, Ana Filipa Cardoso, Filipa Almeida, Olga Azevedo, António Lourenço

**Affiliations:** Department of Cardiology, Unidade Local de Saúde Alto Ave—Hospital da Senhora da Oliveira, Guimarães, Portugal

A 61-year-old man with ischemic heart disease was admitted with acute decompensated heart failure. His disease led to severe left ventricle (LV) systolic dysfunction and the formation of a large basal inferior wall aneurysm with thrombus. Anticoagulation was initiated, and coronary angiography showed severe three-vessel disease. While waiting surgical intervention, the patient had a transient ischemic attack, followed by a left iliac artery embolism, requiring thromboembolectomy. Transthoracic echocardiography revealed a fragmented and mobile intra-aneurysmal thrombus, significantly increasing the embolic risk (Fig. 1 and Supplementary Material, Video S1). Patient underwent successful surgical aneurysm exclusion and coronary artery bypass grafting. This underscores the thromboembolic risk associated with large, mobile LV thrombi, the importance of timely surgical intervention and the impact of LV remodeling in the context of ischemic heart disease [1, 2].

**Fig. 1:**
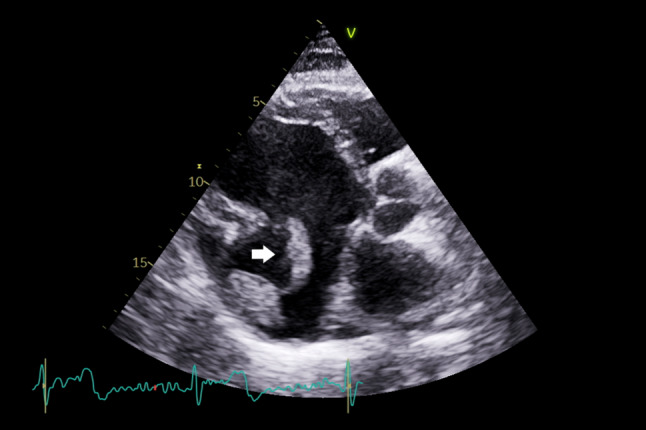
Transthoracic echocardiogram parasternal long-axis view with a posterior tilt showing a fragmented and mobile thrombus (white arrow) within an inferior wall aneurysm

## Supplementary Information

ESM1: Supplementary material 1
